# Design and Implementation of a Dual-Mass MEMS Gyroscope with High Shock Resistance

**DOI:** 10.3390/s18041037

**Published:** 2018-03-30

**Authors:** Yang Gao, Libin Huang, Xukai Ding, Hongsheng Li

**Affiliations:** 1 School of Instrument Science and Engineering, Southeast University, Nanjing 210096, China; gao-yang@seu.edu.cn (Y.G.); huanglibin@seu.edu.cn (L.H.); dingxukai@126.com (X.D.); 2 Key Laboratory of Micro-Inertial Instruments and Advanced Navigation Technology, Ministry of Education, Nanjing 210096, China

**Keywords:** shock resistance, MEMS, micro-gyroscope, dual-mass, shock model

## Abstract

This paper presents the design and implementation of a dual-mass MEMS gyroscope with high shock resistance by improving the in-phase frequency of the gyroscope and by using a two-stage elastic stopper mechanism and proposes a Simulink shock model of the gyroscope equipped with the two-stage stopper mechanism, which is a very efficient method to evaluate the shock resistance of the gyroscope. The structural design takes into account both the mechanical sensitivity and the shock resistance. The design of the primary structure and the analysis of the stopper mechanism are first introduced. Based on the expression of the restoring force of the stopper beam, the analytical shock response model of the gyroscope is obtained. By this model, the shock response of the gyroscope is theoretically analyzed, and the appropriate structural parameters are obtained. Then, the correctness of the model is verified by finite element (FE) analysis, where the contact collision analysis is introduced in detail. The simulation results show that the application of the two-stage elastic stopper mechanism can effectively improve the shock resistance by more than 1900 g and 1500 g in the *x*- and *y*-directions, respectively. Finally, experimental verifications are carried out by using a machete hammer on the micro-gyroscope prototype fabricated by the deep dry silicon on glass (DDSOG) technology. The results show that the shock resistance of the prototype along the *x*-, *y*- and *z*-axes all exceed 10,000 g. Moreover, the output of the gyroscope can return to normal in about 2 s.

## 1. Introduction

With the development of micro-electro-mechanical systems (MEMS) technology, the performances of MEMS devices become greatly improved, and now, these devices are widely used in various fields. As one kind of silicon-based micro-device, micro-gyroscopes have the great advantages of small size, low power consumption, low cost and batch fabrication compared with their conventional counterparts. In the past several decades, with the advancements of the architecture, measuring and controlling technology, micro-gyroscopes have achieved rapid development. At present, they are widely adopted in many fields, including the automotive industry, consumer electronics, robot control systems, aerospace navigation and military weapons [1,2].

Besides the above advantages of MEMS devices, reliability is also a major aspect that determines whether the devices are suitable to be applied or not [3]. The shock loads of applications in some harsh environments such as munition launching from tanks, artillery, and mortar are about 10,000~100,000 g [4,5]. The typical failure modes, which will affect the reliability of MEMS devices, are distinguished in [6], including mechanical fracture, adhesion, charge accumulation, wear, fatigue and creep, electric short circuits and openings and contamination. The failure mode depends on the type of the MEMS devices. According to [3,7], the micro-gyroscopes belong to the category of devices that have moving parts, but without rubbing or impacting surfaces. These devices are particularly susceptible to mechanical shock, which will cause excessive deflections to the movable structural components. Thereby, cracking, chipping and fracture problems caused by the collision or the excessive deformation in the shock environment are the common failure modes of the structure. Besides, stiction and electric short circuits due to the contact between the movable part and the stationary part or the substrate are also the critical sources of failure in micro-scale devices [8]. These effects will deteriorate device reliability, reduce device performance and even damage the device structure [9].

Much effort has already been devoted to improving the shock performance of the MEMS devices, and many achievements have been made. It is noteworthy that the cost of improving shock resistance has to be taken into account. The expenses will increase drastically as the product development moves onward (concept, design, fabrication, assembly, packaging) [10]. Then, for the MEMS devices, the essential method is to take into account the shock reliability at the beginning of the development project (design phase). Simulation and experiment are two main approaches to evaluate the shock resistance of the MEMS. However, the experimental approaches can imply the investigations only after the prototypes are fabricated. Moreover, a second or third redesign might be required for the desired specifications, which will lead to an iterative optimization procedure. Therefore, this approach is expensive and time-consuming. The analyses of simulation approaches are mainly based on the analytical model or the FE model. The FE simulation (the transient dynamic simulation) always takes a long time (always needing several hours). During the structural design, in order to get the desired configuration, the parameters need to be adjusted multiple times, which will take considerable time.

The design phase mainly includes the structural design and the circuit design. It has been widely discussed that the influence of shock is difficult to control by electronics [11]. Hence, it is better to improve the shock resistance of MEMS devices through the structural methods. Enhancing the robustness of the weak structures and using stoppers are two kinds of widely-used methods in MEMS devices to improve the anti-shock performance. Particular structural design for better stress relief is the conventional method to improve the structure robustness, such as the spring corner designs in [12], the circular arc step structure at the corner of a notch structure in [13]. However, this method can only improve the anti-shock performance slightly [14]. Stoppers, including solid stoppers and elastic stoppers, are widely applied in MEMS accelerometers [15,16]. The solid stoppers can limit the displacement caused by shock, but the arising impact forces between the contact components can also induce fractures. While the elastic stoppers can be a good solution to this problem, their effect is limited in comparison with the solid one.

For a dual-mass micro-gyroscope, a useful method is to raise the in-phase mode frequency, which can further improve its anti-shock performance [14,17]. As was studied in our previous work [1], raising the in-phase frequency can improve the impact resistance, while raising the anti-phase frequency will reduce the mechanical sensitivity. As the in-phase mode frequency is close to the anti-phase mode frequency, the increase of the in-phase mode frequency will also cause the increase of the anti-phase mode frequency, which will influence the mechanical sensitivity. Therefore, selecting an appropriate working frequency is very crucial to balance mechanical sensitivity and shock performance.

In this paper, we propose a dual-mass MEMS gyroscope with high shock resistance by improving the in-phase frequency of the gyroscope and using the two-stage elastic stopper mechanism. The structural design takes into account both the mechanical sensitivity and the shock resistance. Besides, we propose a Simulink shock model of the gyroscope equipped with the two-stage stopper mechanism, it is a very efficient method to evaluate the shock resistance of the gyroscope. The correctness of this method is verified by FE analysis. The rest of this paper is organized as follows: Section 2 gives the structural design of the dual-mass MEMS vibratory gyroscope, which includes the primary structure and the two-stage elastic stopper mechanism. The analysis of the two-stage elastic stopper mechanism is briefly introduced, and the expression of the restoring force from the stopper beam is given. The shock response models of the gyroscopes are analyzed in Section 3, which can give guidance to the determination and optimization of structural parameters. The open-loop model is demonstrated first, and the closed-form shock response model for the gyroscope equipped with the two-stage elastic stopper mechanism is obtained. Section 4 presents the transient dynamic analysis by using ANSYS software. The contact collision simulation is introduced in detail. Moreover, the validity of the obtained closed-form shock model and the structural design is verified through the FE simulations. The deep dry silicon on glass (DDSOG) processing technology and the shock experiment using a machete hammer are shown in Section 5. Finally, Section 6 concludes this paper with a summary.

## 2. Structure Design

**Figure 1:**
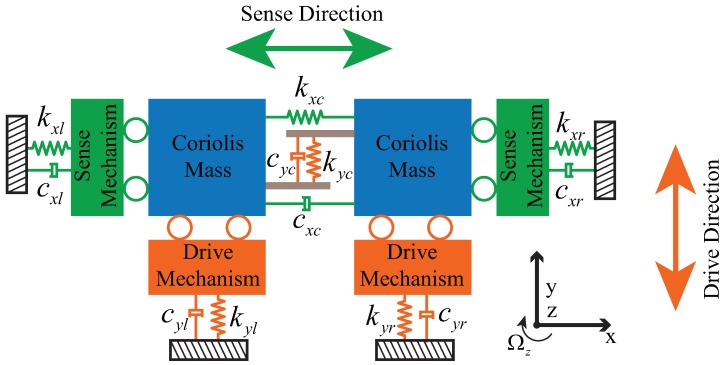
Mechanical model of the dual-mass MEMS gyroscope.

The dual-mass MEMS gyroscope ideal model, a full structure-decoupled model that includes the drive mode and the sense mode, is shown in Figure 1. Each mode has only one degree of freedom (DOF) and can be described as a two-order spring-mass-damping system. In the drive mode, the drive mechanism and the Coriolis mass are driven to oscillate along the drive direction (*y*-axis), and the sense mechanism remains stationary. While in sense mode, the sense mechanism and the proof mass are driven to oscillate along the sense direction (*x*-axis), and the drive mechanism remains stationary. Thus, the drive mode and sense mode are effectively decoupled.

In operation, the two parts of the drive mechanism and the Coriolis masses are driven to anti-phase resonance oscillation along the *y*-axis with the same amplitude. When an angular rate $\Omega_{z}$ is applied, due to the Coriolis effect, anti-phase Coriolis forces will be generated in the two Coriolis masses along the *x*-axis. Therefore, the two parts of the sense mechanism and the Coriolis masses will be actuated into anti-phase vibration with the same rate-related amplitude along the *x*-axis. The input angular rate $\Omega_{z}$ can be obtained by the differential output.

### 2.1. Design of the Primary Structure

**Figure 2:**
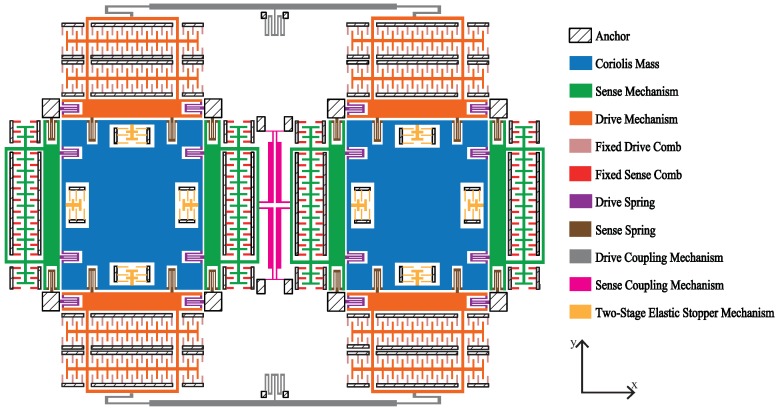
Structural schematic of the dual-mass MEMS gyroscope.

Figure 2 illustrates the whole structure of the dual-mass MEMS gyroscope. As is discussed in detail in [1], the increase of the in-phase mode frequency can reduce the shock sensitivity, but the increase of the anti-phase mode frequency will reduce the mechanical sensitivity. Usually, for the dual-mass gyroscope coupled by elastic beams, the in-phase mode frequency is lower than the anti-phase mode frequency. As a result, the anti-phase mode frequency will also increase with the rising of the in-phase mode frequency. In order to avoid excessive loss of mechanical sensitivity, many researchers have done much work on the coupling mechanisms to achieve modal inversion [1,18,19,20,21]. This paper uses the coupling mechanisms in [18,21] as the drive coupling mechanism and the sense coupling mechanism, respectively.

The drive mode frequency and the sense mode frequency can be given by:

(1) $$f_{d}=\frac{1}{2\pi }\sqrt{\frac{k_{d}+k_{cd}}{m_{d}}}$$

(2) $$f_{s}=\frac{1}{2\pi }\sqrt{\frac{k_{s}+k_{cs}}{m_{s}}}$$

where $k_{d}$ and $k_{s}$ are the stiffness of the support beams along the drive direction and the sense direction, respectively; $k_{cd}$ and $k_{cs}$ are the stiffness of the drive coupling mechanism and the sense coupling mechanism in the anti-phase mode, respectively.

Similarly, the in-phase mode frequencies in the drive direction and the sense direction can be given by:

(3) $$f_{d_{in}}=\frac{1}{2\pi }\sqrt{\frac{k_{d}+k_{cd_{in}}}{m_{d}}}$$

(4) $$f_{s_{in}}=\frac{1}{2\pi }\sqrt{\frac{k_{s}+k_{cs_{in}}}{m_{s}}}$$

where $k_{cd_{in}}$ and $k_{cs_{in}}$ are the stiffness of the drive coupling mechanism and the sense coupling mechanism in the in-phase mode, respectively.

According to Equations (3) and (4), improving the in-phase mode frequency requires reducing the mass or increasing the stiffness. By adjusting the stiffness of the supporting mechanism and the coupling mechanism, as well as the mass of the Coriolis mass, the desired modal configuration is obtained. The main parameters of the structure are shown in Table 1. In this design, square holes in a uniform distribution are dug in the Coriolis mass to improve the in-phase mode frequency, and these holes are replaced by an equivalent large hole in the FE simulation.

**Figure 3:**
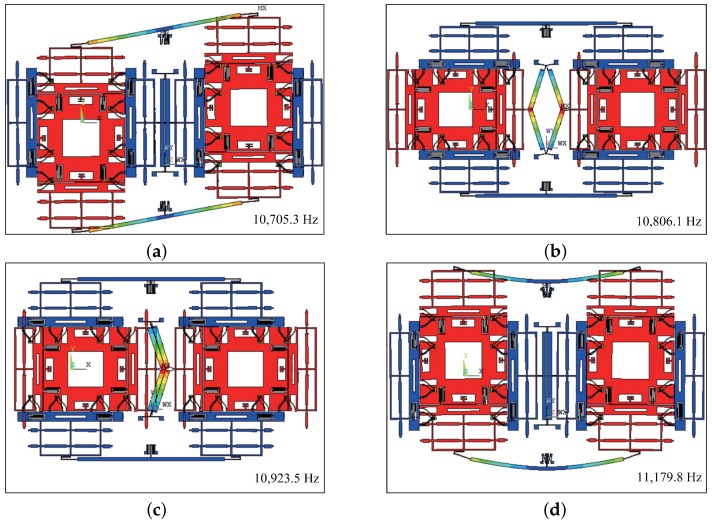
The first four modes of the MEMS gyroscope: (**a**) The anti-phase mode frequency in the drive direction. (**b**) The anti-phase mode frequency in the sense direction. (**c**) The in-phase mode frequency in the sense direction. (**d**) The in-phase mode frequency in the drive direction.

The material properties of silicon are listed in Table 2 [22,23]. Figure 3 shows the first four modes of the dual-mass MEMS gyroscope.

It can be seen that the drive mode frequency and sense mode frequency are 10,705.3 Hz and 10,806.1 Hz, respectively. The in-phase mode frequencies become higher than the anti-phase frequencies in both the drive mode and the sense mode, which means the modes have a reversed change.

### 2.2. Design and Analysis of the Two-Stage Elastic Stopper Mechanism

**Figure 4:**
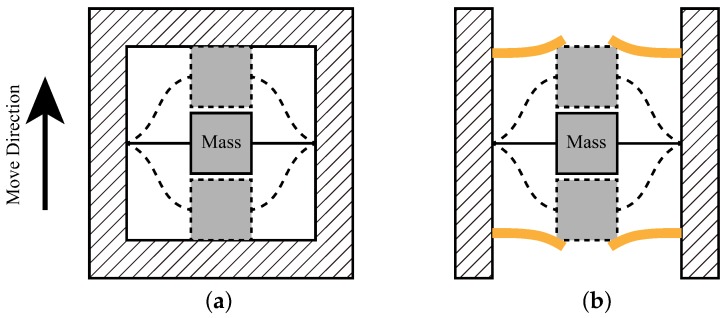
The two types of stoppers: (**a**) The solid stopper. (**b**) The elastic stopper.

In order to further improve the shock resistance of the device, the stopper mechanism is often used. Ideally, the gyroscope only has the degree of freedom along the drive direction and the sense direction. Therefore, the stopper is designed and analyzed for these two directions. As is shown in Figure 4, the stopper can be divided into a solid stopper and elastic stopper [9,14].

**Figure 5:**
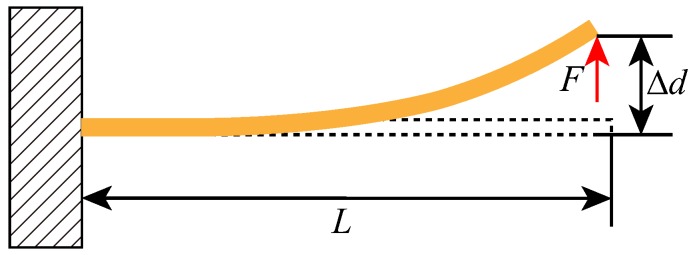
Mechanical analysis of the single clamped cantilever beam.

Figure 5 shows the deformation of the single clamped cantilever beam under the shearing force. According to the relevant theoretical formula [9,24], as the beam deflects, the force (*F*) applied to its tip can be expressed as:

(5) $$F\left(\Delta d\right)=F_{l}+F_{n}=k_{l}\Delta d+k_{n}\Delta d^{3},$$

where $F_{l}$ and $F_{n}$ are the linear and the nonlinear force component; *d* is the displacement of the end of the beam; $k_{l}$ and $k_{n}$ are the linear and the nonlinear stiffness coefficient, which can be expressed as:

(6) $$k_{l}=\frac{3EI}{L^{3}},$$

(7) $$k_{n}=\frac{81EI}{35L^{5}},$$

where *E* is the Young’s modulus of the material; *I* is the moment of inertia; *L* is the length of the beam.

The restoring force from one stopper beam structure can be obtained by $F_{r}=F$, which can be given as:

(8) $$F_{r}\left(\Delta d\right)=k_{l}\Delta d+k_{n}\Delta d^{3}=\frac{3EI}{L^{3}}\Delta d+\frac{81EI}{35L^{5}}\Delta d^{3},$$

**Figure 6:**
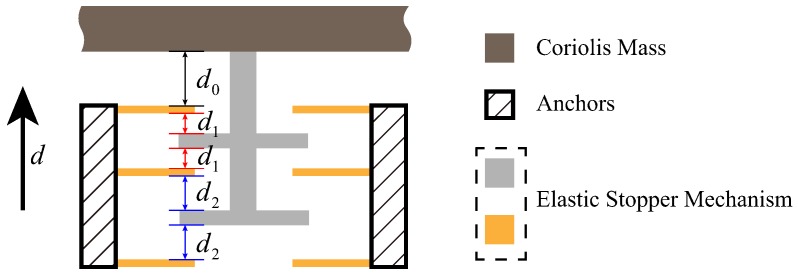
Structural schematic of the two-stage elastic stopper mechanism.

The multi-stage elastic stopper mechanism can realize progressive buffering. The stages of the stopper mechanism can be selected based on practical needs. Based on the structure in [14], as is shown in Figure 6, the two-stage elastic stopper mechanism, which consists of two identical parts, is designed and used. Each part includes three elastic beams connecting to the anchor and two T-shaped frames connecting to the Coriolis mass; where $d_{0}$ represents the maximum allowable displacement of the structure; $d_{1}$, $d_{2}$ are the gaps between the elastic beam and the T-shaped frame in the first-stage and the second-stage elastic stopper mechanism, respectively.

Through Equation (8), the restoring force of the two-stage elastic stopper mechanism can be derived as:

(9) $$F_{r}=\left\{\begin{array}{cc} n_{1}\left[k_{l1}\Delta d_{1}+k_{n1}{\Delta d_{1}}^{3}\right]+n_{2}\left[k_{l2}\Delta d_{2}+k_{n2}{\Delta d_{2}}^{3}\right] & \mathrm{if}\;d>d_{2},\\ n_{1}\left[k_{l1}\Delta d_{1}+k_{n1}{\Delta d_{1}}^{3}\right] & \mathrm{if}\;d_{1}\leq d\leq d_{2},\\ 0 & \mathrm{if}\;-d_{1}\leq d\leq d_{1},\\ -n_{1}[k_{l1}\Delta d_{1}+k_{n1}{\Delta d_{1}}^{3}] & \mathrm{if}\;-d_{2}\leq d\leq -d_{1},\\ -\{n_{1}\left[k_{l1}\Delta d_{1}+k_{n1}{\Delta d_{1}}^{3}\right]+n_{2}\left[k_{l2}\Delta d_{2}+k_{n2}{\Delta d_{2}}^{3}\right]\} & \mathrm{if}\;d<-d_{2}, \end{array}\right.$$

where *d* is the displacement of the Coriolis mass under the shock loads; $n_{1}$, $n_{2}$ are the numbers of the first-stage stopper beams and the second-stage stopper beams, respectively; $\Delta d_{1}$, $\Delta d_{2}$ are the tip displacement of the first-stage stopper beams and the second-stage stopper beams, which can be given by:

(10) $$\Delta d_{1}=\left|d\right|-d_{1}$$

(11) $$\Delta d_{2}=\left|d\right|-d_{2}$$

When shock loads are applied to the device, the elastic stopper mechanism will restrain the movement of the Coriolis mass through the effect of the restoring force.

## 3. Shock Response Model

There are various shock loads in the environments characterized by different frequencies and shapes [25], which can be approximately described by a series of simple shock pulses [4]. In this paper, the half-sine waveform is used for the analysis. According to [26], it can be expressed as:

(12) $$a\left(t\right)=\left\{\begin{array}{cc} A\sin(\omega t) & \mathrm{if}\;0\leq t\leq t_{0},\\ 0 & \mathrm{if}\;t>t_{0}, \end{array}\right.$$

where $\omega =\pi /t_{0}$, $t_{0}$ is the duration time and *A* is the peak acceleration.

For the dual-mass gyroscope without the stopper mechanism, the shock response is analyzed in detail in our previous paper and can be expressed in an analytical form [1]. The kinetic equation under the shock load can be expressed as:

(13) $$\left\{\begin{array}{c} \ddot{x}+\frac{\omega_{x}}{Q_{x}}\dot{x}+{\omega_{x}}^{2}=a,\\ \ddot{y}+\frac{\omega_{y}}{Q_{y}}\dot{y}+{\omega_{y}}^{2}=a, \end{array}\right.$$

where *x*, *y* are the mass displacements in the drive and sense direction; $\omega_{x}$, $\omega_{y}$ are the in-phase mode frequencies along the *x*-axis and *y*-axis; $Q_{x}$, $Q_{y}$ are the quality factors along the *x*-axis and *y*-axis; $m_{x}$, $m_{y}$ are the masses along the *x*-axis and *y*-axis.

**Figure 7:**
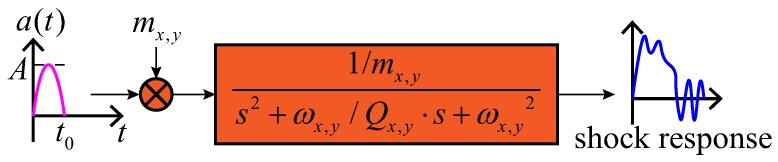
Simulink model of the shock response of the MEMS gyroscope without the stopper mechanism.

Based on Equation (13), the Simulink model of its shock response is obtained, as is shown in Figure 7.

### 3.1. Design and Analysis of the Shock Response Model

For a gyroscope equipped with the stopper mechanism, the shock response is more complicated and is hard to present with an analytic expression. In this section, based on the open-loop model, the shock response model of the MEMS gyroscope equipped with the two-stage elastic stopper mechanism is developed.

Considering the restoring force from the stopper beams, the kinetic equation under the shock load can be expressed as:

(14) $$\left\{\begin{array}{c} \ddot{x}+\frac{\omega_{x}}{Q_{x}}\dot{x}+{\omega_{x}}^{2}=\frac{-F_{rx}+m_{x}a}{m_{x}},\\ \ddot{y}+\frac{\omega_{y}}{Q_{y}}\dot{y}+{\omega_{y}}^{2}=\frac{-F_{ry}+m_{y}a}{m_{y}}, \end{array}\right.$$

where $F_{rx}$, $F_{ry}$ are the restoring forces of the two-stage elastic stopper mechanism along the *x*-axis and *y*-axis, which can be expressed as:

(15) $$F_{rx,ry}=\left\{\begin{array}{cc} \begin{array}{c} n_{1x,1y}\left[k_{l1x,l1y}\Delta d_{1x,1y}+k_{n1x,n1y}{\Delta d_{1x,1y}}^{3}\right]\\ +n_{2x,2y}\left[k_{l2x,l2y}\Delta d_{2x,2y}+k_{n2x,2y}{\Delta d_{2x,2y}}^{3}\right] \end{array} & \mathrm{if}\;d>d_{2x,2y},\\ n_{1x,1y}k_{l1x,l1y}\Delta d_{1x,1y}+k_{n1x,n1y}{\Delta d_{1x,1y}}^{3} & \mathrm{if}\;d_{1x,1y}\leq d\leq d_{2x,2y},\\ 0 & \mathrm{if}\;-d_{1x,1y}\leq d\leq d_{1x,1y},\\ -n_{1x,1y}[k_{l1x,l1y}\Delta d_{1x,1y}+k_{n1x,n1y}{\Delta d_{1x,1y}}^{3}] & \mathrm{if}\;-d_{2x,2y}\leq d\leq -d_{1x,1y},\\ \begin{array}{c} -\{n_{1x,1y}\left[k_{l1x,l1y}\Delta d_{1x,1y}+k_{n1x,n1y}{\Delta d_{1x,1y}}^{3}\right]\\ +n_{2x,2y}\left[k_{l2x,l2y}\Delta d_{2x,2y}+k_{n2x,n2y}{\Delta d_{2x,2y}}^{3}\right]\} \end{array} & \mathrm{if}\;d<-d_{2x,2y}, \end{array}\right.$$

**Figure 8:**
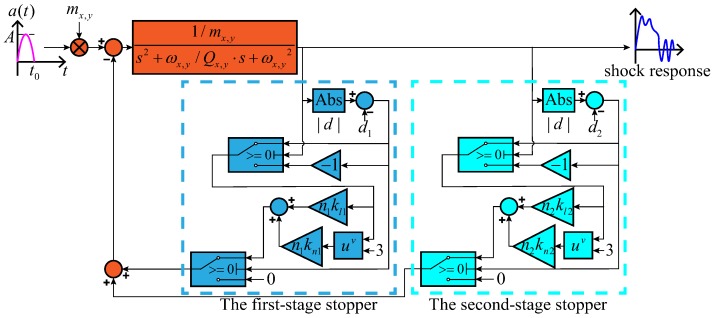
Shock response model of the MEMS gyroscope equipped with the two-stage elastic stopper mechanism.

As both the drive mode and the sense mode can be described as a two-order spring-mass-damping system, the shock response model can be given as one, as is shown in Figure 8.

### 3.2. Simulation of the Shock Response by the Model

For convenience, the model without the stopper mechanism is defined as Model-A, and the one with the two-stage elastic mechanism is defined as Model-B. The length, width and height of the designed stopper beam are 200 μm, 10 μm, 60 μm, respectively. The numbers of the stopper beams $n_{1}$ and $n_{2}$ are both eight. Then, the linear and nonlinear stiffness coefficients can be obtained through Equations (6) and (7). According to our previous experimental results, the quality factor of the MEMS gyroscopes that we developed is about 3000–8000. In this analysis, the quality factor is 5000. The parameters of the simulation model are given in Table 3.

**Figure 9:**
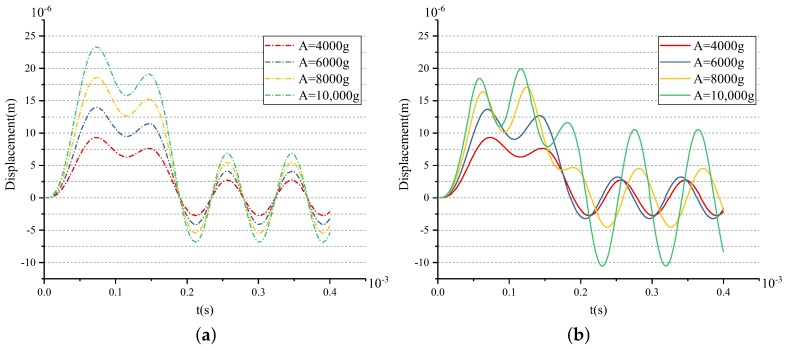
The results of the Simulink model of the shock response of the MEMS gyroscope: (**a**) without the stopper mechanism and (**b**) with the two-stage elastic stopper mechanism.

As the analyses of the drive direction and the sense direction are similar, this subsection focuses on the simulation analysis of the driving direction. Figure 9 gives the results of the shock response under the shock loads with different amplitudes along the drive direction. $d_{1}$ and $d_{2}$ are 12 μm and 14 μm in this group of simulations, respectively. Figure 9a shows the shock response in Model-A, and Figure 9b shows the one in Model-B. It can be seen from the red curves that, under the shock load of 4000 g, the maximum shock displacement is less than $d_{1}$. Therefore, the stopper mechanism does not work under these loads. The shock responses in the two types of model are the same. The blue curves show that the maximum shock displacement is more than $d_{1}$, but less than $d_{2}$ under the 6000 g shock load. In this situation, only the first-stage stopper works, and the maximum shock displacement of Model-B is slightly less than that of Model-A. The yellow and green curves show that both two-stage stoppers can work under the shock loads of 8000 g and 10,000 g. Moreover, the maximum shock displacements of the model with the stopper mechanism are significantly reduced. The displacements are about 91.5% and 85.4% of the model without the stopper mechanism under the shock loads of 8000 g and 10,000 g.

The above results show that the stopper mechanism can effectively improve the shock performance. On the one hand, the stopper mechanism can reduce the displacement caused by massive shock, and on the other hand, it can withstand higher shock load under the same structural admissible gap.

**Figure 10:**
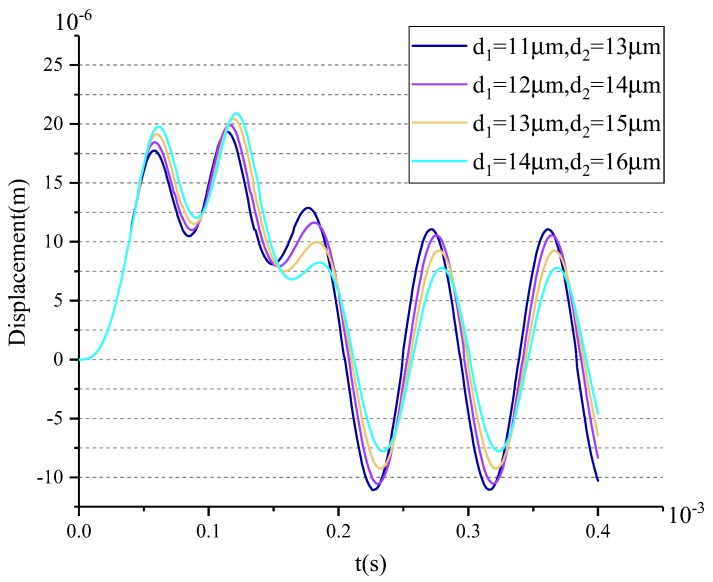
The results of the Simulink model of the shock response of the MEMS gyroscope under different $d_{1}$ and $d_{2}$.

Figure 10 gives the shock responses of Model-B with different $d_{1}$ and $d_{2}$ under the same shock load of 10,000 g.

The results show that the smaller the values of $d_{1}$ and $d_{2}$ are, the smaller the shock responses become. At the same time, the shock force will also be greater. Therefore, the values of $d_{1}$ and $d_{2}$ should be reasonably designed based on the shock response and the shock stress, which are analyzed in detail in the next section. The purple curve in Figure 10 shows that the maximum shock displacement is less than 20 μm (the structural admissible gap) with $d_{1}=12$ μm and $d_{2}=14$ μm. Then, the predetermined values of $d_{1}$ and $d_{2}$ are 12 μm and 14 μm based on the target of anti-10,000 g shock. Similarly, predetermined values of the gaps in the stopper along the sense direction can be obtained, which are 11 μm and 13 μm, respectively.

## 4. Transient Dynamic Analysis

**Figure 11:**
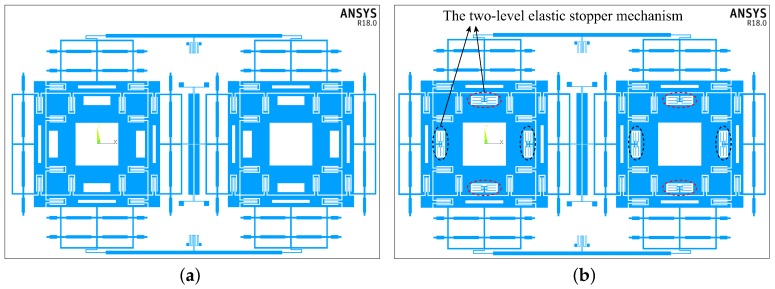
The structure model for FE simulation: (**a**) The structure without stopper mechanism. (**b**) The structure with the two-stage elastic stopper mechanism.

Transient dynamic analyses of the two structures are conducted by using the ANSYS/LS-DYNA software. The shock response and the shock stress in the structure can be obtained by the simulation. Figure 11 shows the two types of simulation models of the structures. Figure 11a shows the structure without the stopper mechanism, and Figure 11b shows the one with the two-stage elastic stopper mechanism. The simulation parameters are shown in the above Table 1, Table 2 and Table 3.

The full method, reduced method and model superposition method are three methods for the transient dynamics analysis. The full method allowing the inclusion of various non-linear properties (plasticity, large deformation, considerable strain) uses the full system matrix to calculate the transient response and is the strongest of the three methods [27]. This paper uses the full method in the transient dynamics analysis.

Besides, the damping effect is crucial to determine the dynamic transient response. As one of the most widely-used damping models, the Rayleigh damping model is used in this paper. The determination method of Rayleigh damping constants α and β was described in detail in our previous article [1].

**Figure 12:**
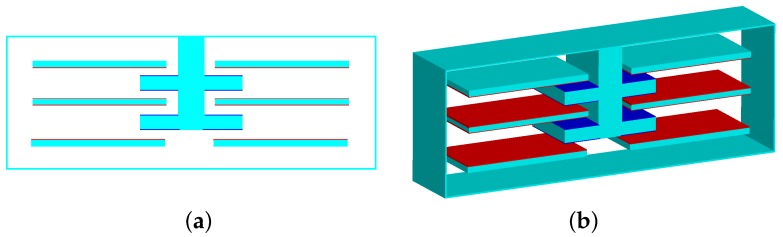
The surface-to-surface contact: (**a**) the front view (2D). (**b**) the three-dimensional map (3D).

In the analysis of the structure with the two-stage elastic stopper mechanism, the contact conditions need to be set. In this situation, the surface-to-surface contact is used, as is shown in Figure 12. The red component and the blue component are the target surface and the contact surface, respectively, which form the contact pair.

**Figure 13:**
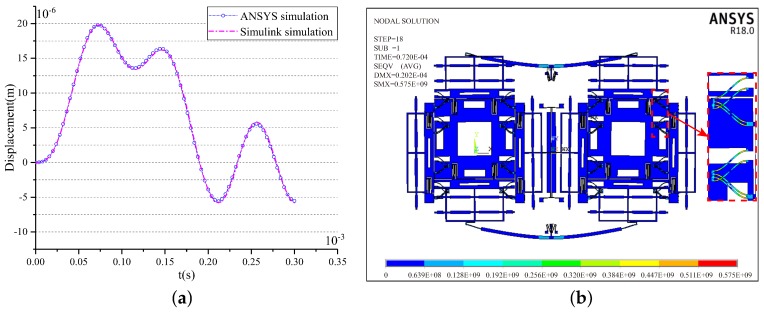
The shock response and the stress of the structure without the stopper mechanism under 8500 g shock load along the *y*-axis.

Firstly, the limited shock load of Structure-A along the drive direction is analyzed. The results of Simulink simulation and the ANSYS simulation are shown in Figure 13a. It can be seen that the two simulation results coincide well with each other. The slight difference between them is mainly caused by the equivalent treatments adopted in model simplification and the unsymmetrical meshing in simulation modeling. The results show that the limited shock load is 8500 g as the permissible gap of the structure is 20 μm. The maximum shock displacement is 19.80 μm at 0.076 ms by the ANSYS simulation and is 19.81 μm at 0.076 ms by the Simulink simulation under the limit shock load. Figure 13b shows the stress distributions at the maximum shock displacement. It is evident that the stress is far lower than the fracture strength of silicon materials (790 MPa).

When the shock load increases, the maximum shock displacement will exceed the allowable gap of the structure, which will cause the failure in MEMS by stiction and electric short circuits due to the contact between the movable part and the stationary part.

**Figure 14:**
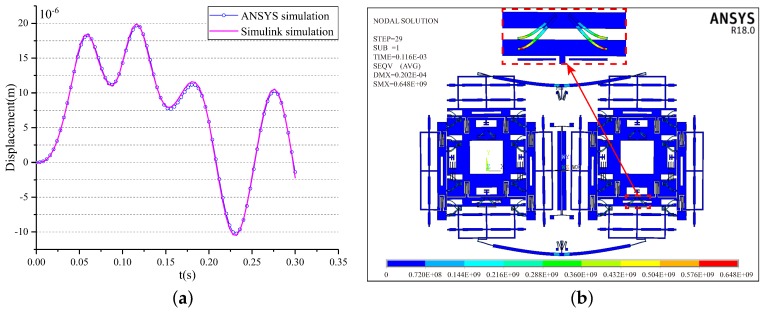
The shock response and the stress of the structure with the two-stage elastic stopper mechanism under 10,000 g shock load along the *y*-axis.

Then, the limited shock load of Structure-B along the drive direction is analyzed. Figure 14a illustrates the results of Simulink simulation and the ANSYS simulation under the 10,000 g shock load. The two simulation results also coincide well with each other. The maximum shock displacement is 19.68 μm at 0.076 ms by the ANSYS simulation and is 19.91 μm at 0.076 ms by the Simulink simulation under the limited shock load, which are both lower than the permissible gap of the structure (20 μm). Figure 14b shows the stress distributions at the maximum shock displacement. The magnified picture in Figure 14b shows that the maximum stress is located at the stopper spring and is 648 MPa, which is far lower than the fracture strength of silicon materials. Therefore, Structure-B can withstand a 10,000 g shock load.

Figure 13 and Figure 14 show that the shock resistance ability along the driving direction has increased from 8500 g–10,000 g by using the two-stage elastic stopper mechanism. The results of the shock displacement and the stress show that the selected values: $d_{1}=12$ μm and $d_{2}=14$ μm are suitable.

The transient dynamic analysis along the sense direction is similar to that along the drive direction. Ideally, there is no degree of freedom along the *z*-axis. However, in fact, there will still be a displacement under the high shock load, which is analyzed by the same method. The simulation results of the transient dynamic analysis along the *x*-, *y*- and *z*-axis are given in Table 4, which show that the structure with the two-stage elastic mechanism can withstand the 10,000 g shock load in all the *x*-, *y*- and *z*-directions.

## 5. Processing and Experiment

### 5.1. Processing and Packaging

**Figure 15:**
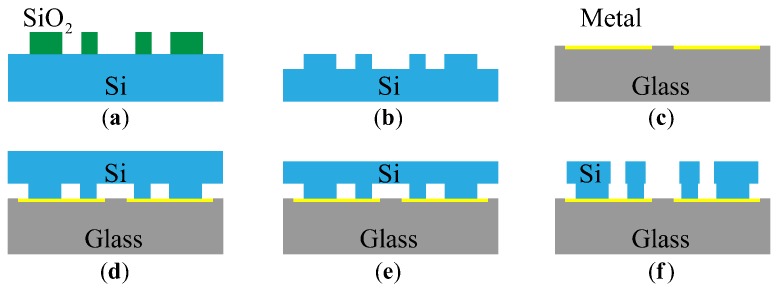
The procedure of the deep dry silicon on glass (DDSOG) technology. (**a**) Anchoring on the silicon wafer. (**b**) Etching the bonding area on the silicon wafer. (**c**) Depositing the metal conductive layer onto the glass substrate. (**d**) Bonding the silicon wafer to the glass substrate. (**e**) Thinning and polishing the silicon wafer. (**f**) Inductively coupled plasma (ICP) etching and structure releasing.

The micromechanical resonant output gyroscope was fabricated by using the DDSOG technique. The materials used in structural layer and substrate of the device are silicon and glass, respectively. Figure 15 illustrates the fabrication process.

**Figure 16:**
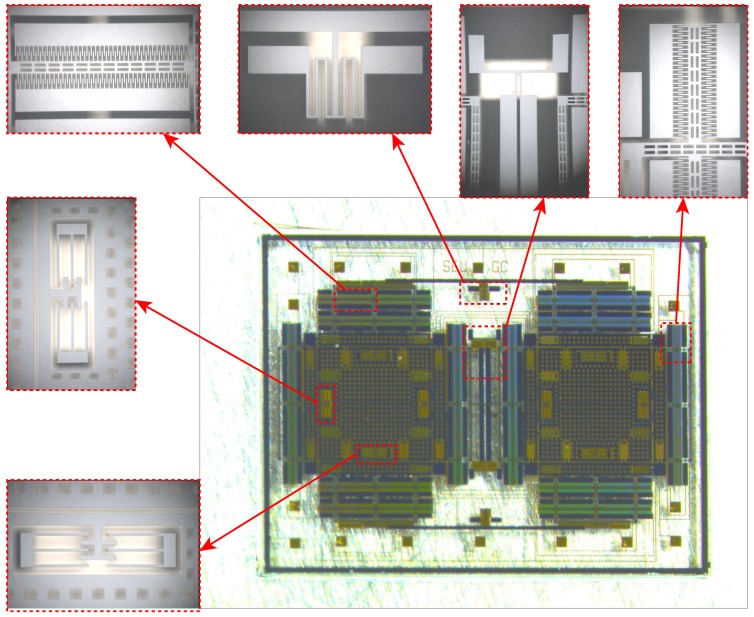
The SEM photos of the structure.

The fabricated structure of the micro-gyroscope equipped with the two-stage elastic stopper mechanism is shown in Figure 16, and the SEM photos of several essential parts are also given.

**Figure 17:**
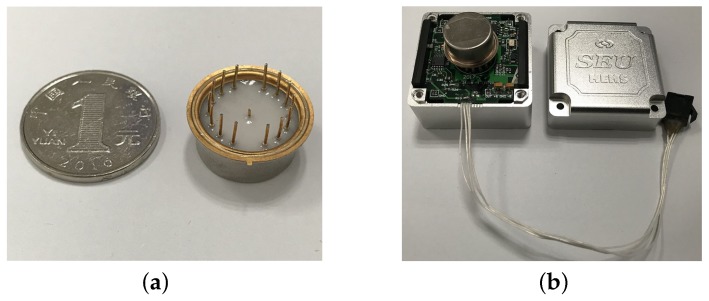
(**a**) The vacuum packaged gyroscope. (**b**) The gyroscope with the circuit.

The prototype is vacuum packaged, as is shown in Figure 17a. Figure 17b gives the device with the circuit.

### 5.2. Experiments

**Figure 18:**
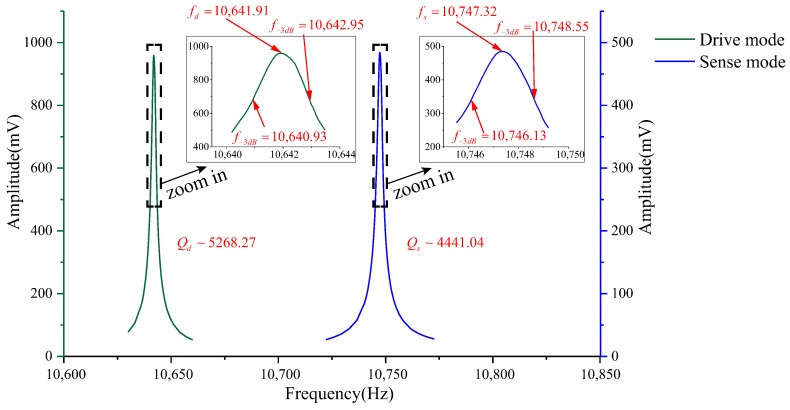
Experimental input-output amplitude-frequency responses of the gyroscope.

A 5-V DC and a 2-V AC are applied on the driving electrode of the gyroscope. The frequency of the AC voltage is swept from 10,530 Hz–10,560 Hz by using the waveform generator. The electrostatic force will actuate the active structure to vibrate. When the frequency of AC voltage comes to the drive-mode frequency, the gyroscope will reach the full resonant state, then the maximum value of vibration amplitude of active microstructure can be observed. Similarly, the input-output amplitude-frequency response of the sense mode is carried out. Figure 18 gives the frequency sweeping experiment results of the drive mode and the sense mode. The results show that the frequencies of the drive mode and the sense mode of the prototype are 10,641.91 Hz and 10,747.32 Hz with quality factors of 5268.3 and 4441.0, which are 0.59% and 0.54% lower than the design values, respectively.

As is well known, over-etching will reduce the width of flexure beams, which causes the reduction of the resonant frequency; conversely, under-etching will lead to the increase of the frequency. The main reason resulting in the slight difference between measured and analytical values of resonant frequencies is fabrication imperfections. Besides, the slight mismatch between material parameters of simulation and actual values, as well as the test error are other factors affecting the accuracy and the compatibility of test and design. However, on the whole, the test results are well consistent with the design values.

**Figure 19:**
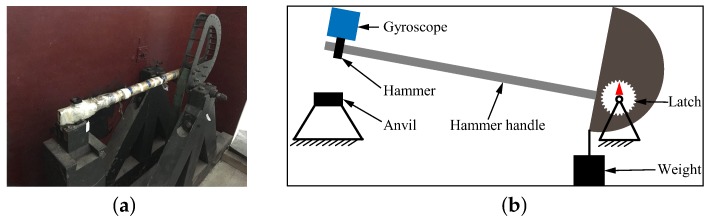
(**a**) Test device (machete hammer); (**b**) schematic for the dynamic experiment of the machete hammer.

A machete hammer is used for the shock experiment. Figure 19a gives the test device, and Figure 19b illustrates the schematic of the dynamic experiment. The hammer is fixed on the handle, while the handle is fixed on the curved turn. The measured gyroscope is fixed on the special fixture, which will then be installed in the hammer. The belt would pull the weight up when the arm rotates. A different number of rotating teeth will lead to different corresponding shock acceleration.

The shock platform is opened after the gyroscope output is stable, and the static data of 10 min are collected. Then, the shock load with a duration of 0.2 ms is acted on the gyroscope. After the shock, the static data of 10 min are also collected. There is a half-an-hour interval between the experiments in the direction of *X*, *Y* and *Z*. According to the instructions of the machete hammer, 12 teeth are rotated in the experiment, which can bring a 9713 ± 396 g shock load. The experimental results obtained in the directions of *x*, *y* and *z* are listed in Table 5.

Table 5 illustrates that the output of the gyroscope will increase rapidly during the shock process and will return to stable in a few seconds. The results show that, not only does the gyroscope suffer no damage under this shock load, but it can also get back to normal work in about 2 s. The average values of the zero bias in the form of voltage before and after the shock are shown in Table 6.

As the experimental scale factor of the gyroscope is 11.36 mV/(deg/s), the variation of the average zero bias under the shock of 10,000 g along the *x*-, *y*- and *z*-directions is −0.166 deg/s, 0.012 deg/s and 0.102 deg/s, respectively. It can be seen that the variation of the average zero bias is more susceptible to the shock along the *x* direction among the three directions. The main reason is that the direction of the detection is the *x* direction. Besides, the shock in the *z*-axis has also a heavy influence on the variation of the average zero bias, and this is because the stiffness in the *z* direction is not large enough. The shock in the *y*-axis has the smallest impact on the variation of the average zero bias among three directions, which shows that the drive direction and sense direction have a good decoupling effect.

These results show that all the shock resistances of the prototype along the *x*-, *y*- and *z*-axes exceed 10,000 g, which can verify the correctness of the design and analysis of the gyroscope.

## 6. Conclusions

The micro-gyroscope is an important part of MEMS inertial sensors. Shock resistance is one of the primary issues in the design of silicon micro-gyroscopes in many applications and should be taken into account at the begging of the development project (design phase). As the in-phase mode frequency is close to the anti-phase mode frequency, while raising the in-phase mode frequency to improve the shock resistance, the anti-phase mode frequency will also be increased, which will reduce the mechanical sensitivity.

In this paper, we have presented the design and implementation of a dual-mass MEMS gyroscope with high shock resistance by improving the in-phase frequency of the gyroscope and by using the two-stage elastic stopper mechanism. The shock resistance of the structure is verified by experiment. Both the mechanical sensitivity and the shock resistance have been taken into account in the structural design. Besides, a Simulink shock model of the gyroscope equipped with the two-stage stopper mechanism is also proposed, which is a very efficient method to evaluate the shock resistance of the gyroscope. The correctness of this method is verified by FE analysis.

Firstly, the working frequency is adjusted to be 10 kHz by modifying the structural stiffness and the mass. Then, the restoring force model of the single clamped cantilever beam under the shearing force is studied. Based on this model, the analysis of the two-stage elastic stopper mechanism is introduced, and the analytical shock response model of the gyroscope with the stopper mechanism is developed, the validity of which has been verified through the FE simulations. The stop effects of the two-stage elastic stopper mechanism under different gaps are analyzed. Analysis results show that the smaller the gap is, the better the stop effect becomes. According to the results of the shock displacement and the stress, the gaps of the two-stage elastic stopper mechanism are 12 μm and 14 μm along the driving direction, as well as 11 μm and 13 μm along the sensing direction. The simulation results show that the application of the two-stage elastic stopper mechanism can effectively improve the shock resistance by more than 1900 g and 1500 g in the *x*- and *y*-directions, respectively. Finally, the processing technology is introduced briefly, and the frequency sweeping experiment of a prototype is conducted. The results show that the frequencies of the drive mode and the sense mode are 10,641.91 Hz and 10,747.32 Hz, 0.59% and 0.54% lower than the design values, respectively. The shock experiments by using a machete hammer show that the shock resistance of the prototype along the *x*-, *y*- and *z*-axes are all superior to 10,000 g. Moreover, the output of the gyroscope can quickly return to normal in about 2 s. These results verify the correctness of the design and simulation.

As an efficient and economical approach, the shock response model is a very beneficial and convenient method to evaluate the shock resistance of the device. This paper developed the model of the two-stage elastic stopper mechanism, which can further provide a reference for the design and application of the single-stage or multi-stage stopper mechanism. The future work mainly includes the influence of structural shock displacements along the *x*-, *y*- and *z*-axes on the driving capacitance and the sensing capacitance and the influence mechanism of the shock load on the detection output signal.

## Figures and Tables

**Table 1:** The main parameters of the structure.

Parameters	Values
Overall dimensions of the whole structure (μm${}^{3}$)	7200×4400×60
Overall dimensions of the Coriolis mass (μm${}^{3}$)	2000×2000×60
Length of the drive spring (μm)	280
Width of the drive spring (μm)	9
Length of the sense spring (μm)	285
Width of the sense spring (μm)	9
Length of the drive coupling leverage (μm)	3496
Width of the drive coupling leverage (μm)	75
Length of the sense coupling leverage (μm)	1000
Width of the sense coupling leverage (μm)	100

**Table 2:** Material parameters for FE simulations.

Parameters	Young’s Modulus (Pa)	Poisson’s Ratio	Density (kg/m${}^{3}$)
Values	$1.7\times 10^{11}$	0.28	2330

**Table 3:** The parameters of the shock model.

$m_{x}$ (kg)	$m_{y}$ (kg)	$k_{l1},k_{l2}$ (N/m)	$k_{n1},k_{n2}$ (N/m)	$\omega_{x}$ (rad/s)	$\omega_{y}$ (rad/s)	$Q_{x},Q_{y}$
$1.0296\times 10^{-6}$	$1.0765\times 10^{-6}$	519.03	$1.385\times 10^{10}$	2·π·10,923.5	2·π·11,179.8	5000

**Table 4:** Simulation results of the transient dynamic analysis along the *x*-, *y*- and *z*-axis.

Parameters		*x*-Axis		*y*-Axis		*z*-Axis	
Stopper		without	with	without	with	-	
Shock load (g)		8100	10,000	8500	10,000	10,000
Maximum displacement (μm)	Simulink simulation	19.74	19.78	19.81	19.68	-
	ANSYS simulation	19.87	19.90	19.80	19.91	3.71
Maximum stress (MPa)		643	752	575	648	281

**Table 5:** The shock experiment results.

	Before	In-Shock	After
*X*	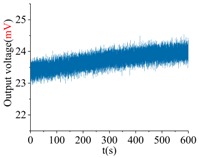	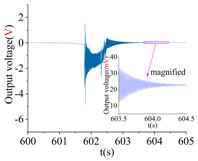	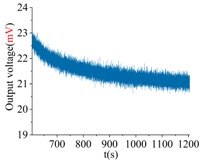
*Y*	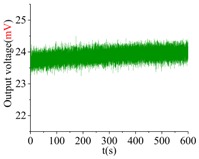	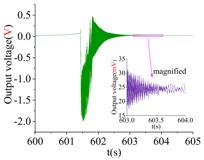	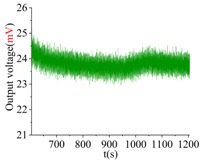
*Z*	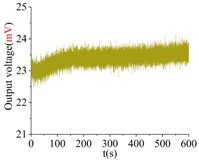	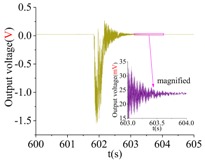	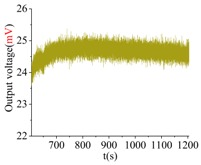

**Table 6:** Simulation results of the transient dynamic analysis along the *x*-, *y*- and *z*-axis.

Shock Direction	*X*		*Y*		*Z*	
**Time Period**	**Before**	**After**	**Before**	**After**	**Before**	**After**
Average zero bias (mV)	23.51	21.62	23.82	23.96	23.35	24.51
